# External Ventricular Drain Management in Aneurysmal Subarachnoid Hemorrhage: Perspectives and Practices From Low-Income and Middle-Income Countries

**DOI:** 10.7759/cureus.83265

**Published:** 2025-04-30

**Authors:** Abhijit V Lele, Hemanshu Prabhakar, Victor Lin, Indu Kapoor, Charu Mahajan, Umeshkumar Athiraman, Nophanan Chaikittisilpa, Ananya Abate Shiferaw, Jorge H Mejia-Mantilla, Peter Tan, Samuel Blacker

**Affiliations:** 1 Neurocritical Care and Anesthesiology, Harborview Medical Center, Seattle, USA; 2 Neuroanaesthesiology and Neurocritical Care, All India Institute of Medical Sciences, New Delhi, New Delhi, IND; 3 Neurology, Harborview Medical Center, Seattle, USA; 4 Anesthesiology, Washington University School of Medicine, St. Louis, USA; 5 Anesthesiology, Mahidol University, Bangkok, THA; 6 Anesthesiology, Addis Ababa University, Addis Ababa, ETH; 7 Neurointensive Care, Fundación Valle del Lili, University Hospital, Cali, COL; 8 Anesthesiology and Intensive Care, Sarawak General Hospital, Kuching, MYS; 9 Anesthesiology, University of North Carolina at Chapel Hill, Chapel Hill, USA

**Keywords:** aneurysm, evd, guidelines, subarachnoid hemorrhage, transport, ventriculostomy, xternal ventricular drain

## Abstract

Objective: This study aimed to evaluate the current practices, challenges, and quality improvement (QI) opportunities related to external ventricular drain (EVD) care in aneurysmal subarachnoid hemorrhage (aSAH) across low- and middle-income countries (LMICs). The findings were compared to international guidelines, including those by the American Heart Association (AHA), Neurocritical Care Society (NCS), and Society for Neuroscience in Anesthesiology and Critical Care (SNACC).

Methods: A cross-sectional survey was conducted between September and December 2024 using a 57-item questionnaire distributed to healthcare providers in LMICs. Data on EVD insertion and management, infection prevention, intracranial pressure (ICP) monitoring, transport practices, and QI metrics were analyzed descriptively, with thematic analysis of free-text responses.

Results: Complete responses were received from 89 participants across 24 countries. Hydrocephalus was the primary indication for EVD insertion (96%), performed mainly in operating rooms (96%) by attending neurosurgeons (73%). Infection-related metrics were reported by 71% of respondents. Key areas for improvement included education, infection prevention, and standardized protocols. The adherence to AHA/NCS/SNACC recommendations was as follows: hydrocephalus as an indication for EVD insertion (96%), sterile technique (91%), EVD clamp trials (81%), indication-based CSF sampling (67%), pre-procedure antibiotics (61%), tunneling catheter (40%), use of anti-microbial-impregnated EVD (17%), and ICP during patient transport (13%).

Conclusions: Significant gaps in EVD care in LMICs highlight the need for tailored QI initiatives. Leveraging campaigns like the SNACC EVD Safety Campaign can drive education, standardization, and improved outcomes. Future efforts should focus on context-specific guidelines and scalable QI practices in resource-limited settings.

## Introduction

The global incidence of aneurysmal subarachnoid hemorrhage (aSAH) ranges from 0.71 to 12.38 per 100,000 persons [1]. Despite advances in medical care, aSAH remains a critical public health issue, particularly in low- and middle-income countries (LMICs), where healthcare resources and access to specialized care may be limited [2,3]. The management of aSAH is multifaceted, requiring prompt treatment of the underlying aneurysm and the management of complications such as hydrocephalus, which occurs in up to 85% of aSAH cases. External ventricular drains (EVDs) are critical for managing hydrocephalus and controlling elevated intracranial pressure (ICP). However, using EVDs effectively demands adherence to quality and safety protocols to minimize complications such as infections, hemorrhages, and device misplacement.

This study focuses on two interrelated aspects of aSAH management: evaluating current practices for managing hydrocephalus in patients with aSAH in LMICs and conducting an in-depth assessment of the quality and safety of EVD use in these settings. Adherence to international guidelines, including those from the American Heart Association (AHA), the Neurocritical Care Society (NCS), and the Society for Neuroscience in Anesthesiology and Critical Care (SNACC), is central to this evaluation. The AHA guidelines [4] emphasize the crucial role of early and appropriate EVD care in managing patients with aSAH, particularly those with hydrocephalus and elevated ICP. According to the guidelines, prompt insertion of EVD and meticulous management can mitigate complications like cerebral herniation and delayed cerebral ischemia. The AHA stresses the importance of using sterile techniques, routine infection control measures, and the clamping of EVDs before considering permanent CSF diversion. These measures are essential for reducing morbidity and improving outcomes in patients with aSAH. The NCS recommendations for EVDs emphasize the importance of an EVD bundle and steps taken to reduce the rates of EVD-associated infections [5]. The SNACC guidelines underscore the significance of EVD care during intrahospital transport [6].

The primary aims of this study are to benchmark current practices against these guidelines, identify areas of concordance and divergence, and propose actionable strategies to enhance the safety and quality of EVD use in resource-constrained settings. By understanding gaps in adherence and the unique challenges faced in LMICs, this study seeks to inform context-specific improvements in aSAH management and contribute to developing global standards.

## Materials and methods

This study was conducted at Harborview Medical Center, University of Washington, Seattle. The University of Washington's Institutional Review Board reviewed and approved this study (STUDY00021382) on 09/23/2024.

Study design

A cross-sectional, survey-based study was conducted using a structured electronic questionnaire to healthcare providers in LMICs. The survey questions were designed based on the 2023 AHA aSAH guidelines, alongside recommendations from the NCS and SNACC on managing EVDs.

Survey study participants

The target participants include neurosurgeons, intensivists, and advanced practice providers who manage aSAH patients requiring EVDs. They were recruited from LMIC hospitals based on the contacts list in the PRINCE LMIC study [7].

Survey instrument

A 57-question survey was designed using REDCap [8]. The link to the survey is as follows: https://redcap.link/EVDLMIC. The survey questions covered decision-making for EVD insertion (AHA recommendations), EVD insertion and maintenance bundle (NCS, SNACC recommendations), protocols for monitoring ICP and preventing infections (NCS recommendations), practice for clamping and removal of EVD (AHA recommendations), intrahospital transport (SNACC recommendations), and explored the challenges faced in implementing guidelines in LMIC hospitals. The survey was reviewed by senior neurosurgeons and intensivists from LMICs who were not involved in the study.

Survey distribution

The survey was emailed between September 23, 2024, and December 5, 2024, and reminders were sent weekly. No financial incentive was provided for participation in the study. Participation was purely voluntary, and participants consented to publish the survey results.

Data analysis

Descriptive statistics were used to summarize the demographic distribution of survey respondents, hospital characteristics, and EVD practices. Categorical variables were presented as frequencies and percentages. Comparisons of survey responses across WHO regions and income groups were performed using Pearson’s chi-square (χ²) test or Fisher’s exact test for categorical variables, depending on sample size and expected cell counts. Statistical comparisons included key variables such as timing of EVD insertion, ICP monitoring during transport, use of antimicrobial-impregnated EVDs, and prophylactic antibiotic administration across different WHO regions. For free-text responses, qualitative data were analyzed thematically. Recurring themes were identified using inductive coding, and the frequency of each theme was reported. Responses related to barriers to guideline adherence, alternative drainage methods, and quality improvement (QI) initiatives were categorized accordingly. A heatmap was generated to visualize adherence to published guidelines across different EVD practices. Adherence rates were calculated as the proportion of respondents reporting each practice. For cost analysis, reported EVD and cerebrospinal fluid (CSF) collection system costs were presented. All statistical analyses were conducted using R Studio [9] (version 4.4.0, Posit Software, Boston, MA), utilizing the tidyverse, ggplot2, and gtsummary packages for data visualization and summary tables. Heat maps were generated using Python (Python Software Foundation, Fredericksburg, VA). A p-value of <0.05 was considered to be statistically significant.

## Results

Survey overview

**Figure 1:**
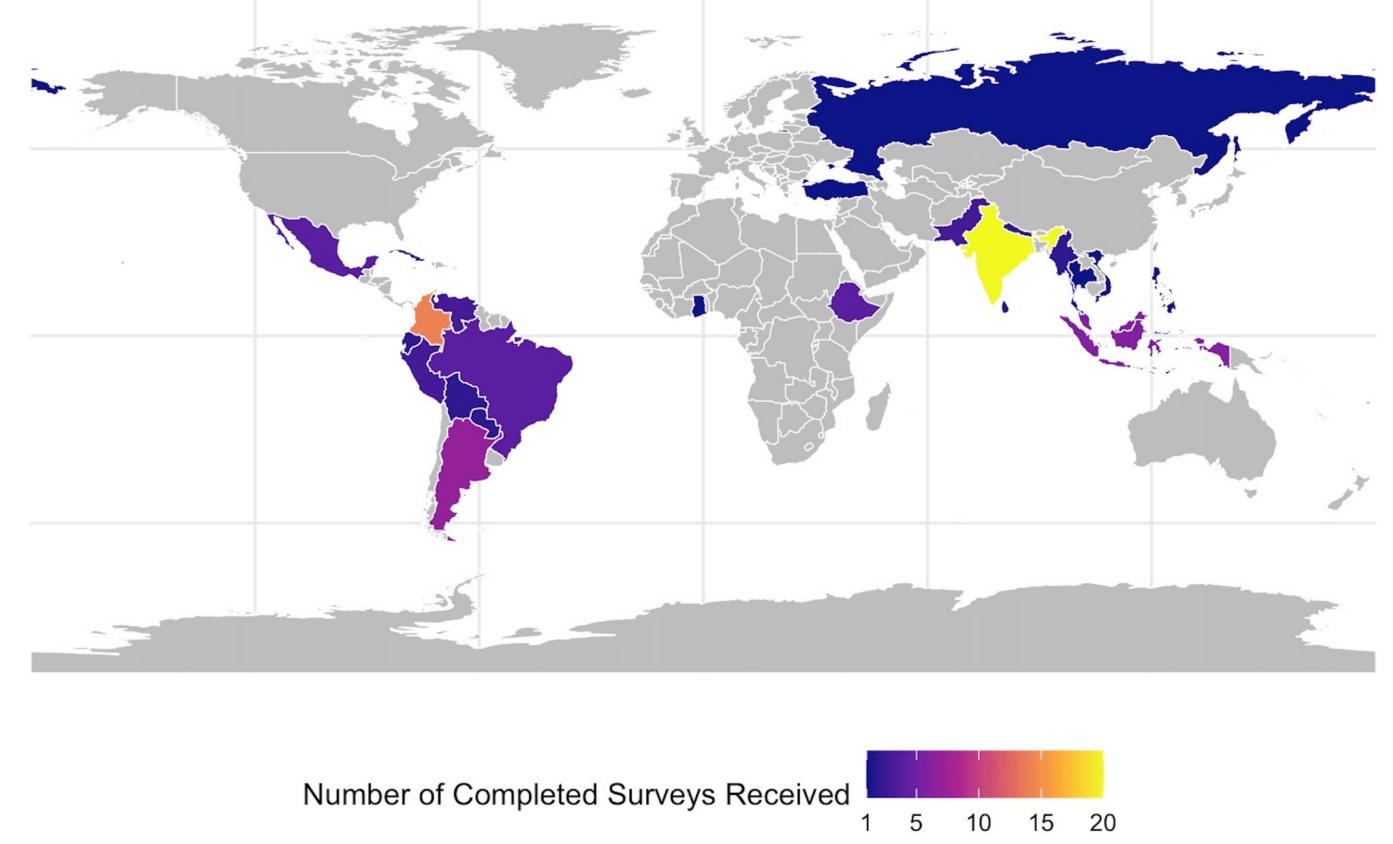
World map with EVD-SAH-LMIC survey participants The figure was created using RStudio. EVD: external ventricular drain; SAH: aneurysmal subarachnoid hemorrhage; LMIC: low or middle-income countries

The survey was sent to 402 recipients and was completed by 89 (22% response rate) representing 24 countries spanning five WHO regions, with the highest contributions from South Asia (India: 20, Pakistan: 3, Nepal: 2, Sri Lanka: 1) and Latin America & the Caribbean (Colombia: 14, Argentina: 7, Mexico: 4, Brazil: 3, among others). Other notable contributions came from East Asia & the Pacific (Indonesia: 6, Malaysia: 6, Thailand: 4), Sub-Saharan Africa (Ethiopia: 4, Ghana: 1), and Europe & Central Asia (Turkey: 1, Russia: 1) (Figure 1). Government teaching hospitals comprised the largest group with 51% (n=45). Most respondents were critical care specialists (63%, n=57). Regarding annual aSAH patient admission rates, 46% (n=41) of facilities admitted between one and 30 patients, 36% (n=32) admitted between 31 and 60 patients, and 18% (n=16) admitted more than 60 patients annually (Table 1).

**Table 1:** Characteristics of participating hospitals and admission characteristics

Characteristics	Count (percentages) n=89
Regions	
East Asia & Pacific	19 (21%)
Europe & Central Asia	2 (2%)
Latin America & Caribbean	38 (43%)
South Asia	25 (28%)
Sub-Saharan Africa	5 (6%)
Income groups	
Low-income country	4 (4%)
Lower-middle-income country	31 (35%)
Upper-middle income country	54 (61%)
Type of hospital	
Government teaching	45 (51%)
Government non-teaching	5 (6%)
Corporate teaching	26 (29%)
Corporate non-teaching	13 (15%)
Survey participant	
Critical care specialist	57 (63%)
Neurosurgeon	15 (16%)
Anesthesiologist	7 (8%)
Neurologist	5 (6%)
Neuroanesthesiologist	4 (4%)
Nurse	1 (1%)
Number of patients with aneurysmal subarachnoid hemorrhage admitted annually	
1-30	41 (46%)
31-60	32 (36%)
>60	16 (18%)
Number of external ventricular drains placed yearly at the hospital	
1-9	12 (13%)
10-50	27 (53%)
51-100	20 (22%)
>100	10 (11%)

External ventricular drain insertion practices

The factors and practices related to EVD insertion are summarized in Table 2. The majority, 96% (n=85), considered the presence of hydrocephalus crucial for assessing the need for EVD insertion. Other factors included Fisher or modified Fisher score in 42% (n=37), Hunt and Hess grade in 29% (n=26), World Federation of Neurological Societies grade in 28% (n=25), and aneurysm location in 12% (n=11). Regarding the timing of EVD insertion, 57% (n=51) preferred to insert the EVD as soon as the diagnosis was made, 24% (n=21) delayed after securement of the aneurysm, and 19% (n=17) inserted it in the operating room just before the intervention.

**Table 2:** External ventricular drain (EVD) insertion practices, as reported by survey participants

Questions	Responses count (percentages) n=89
In patients with aSAH, what factors are considered to assess the need for EVD insertion?	
Presence of hydrocephalus	85 (96%)
Hunt and Hess grade	26 (29%)
World Federation of Neurological Societies grade	25 (28%)
Fisher or modified Fisher score	37 (42%)
Aneurysm location	11 (12%)
Which one of the following is the preferred time to insert EVD in aSAH patients at your hospital?	
As soon as the diagnosis is made	51 (57%)
Delayed after the securement of the aneurysm	21 (24%)
In the operating room, just before the start of the intervention	17 (19%)
Who typically inserts EVDs at your institution? Check all that may apply.	
Resident in a neurosurgery training program	42 (47%)
Fellow in a neurosurgery training program	20 (22%)
Attending neurosurgeon	64 (72%)
Please describe the location in the hospital where EVDs are inserted (check all that may apply).	
Operating room	85 (96%)
Intensive care unit	25 (28%)
Emergency room	8 (9%)
Floor/wards	1 (1%)
Patients with EVD are routinely cared for	
Dedicated neurocritical care unit	36 (40%)
General intensive care unit	49 (55%)
On the floor/wards	4 (4%)
What is the prevalent technique for securing a ruptured intracerebral aneurysm?	
Microsurgical repair	47 (53%)
Endovascular repair	42 (47%)
Timing of the type of aneurysm repair may determine the timing of the EVD insertion	56 (63%)
Techniques used to insert external ventricular drains	
Freehand technique	74 (83%)
Computerized tomography-guided	22 (25%)
Ultrasound-guided	8 (9%)

EVD insertions were typically performed by attending neurosurgeons in 72% (n=64) of cases, with residents in a neurosurgery training program involved in 47% (n=42) and fellows in 22% (n=20). EVDs were most commonly inserted in the operating room (96%, n=85), with some (28%) insertions in the intensive care unit (n=25) and rarely (9%) in the emergency room (n=8) or on the floor/wards (1%, n=1). Patients with EVD were predominantly cared for in general intensive care units (55%, n=49) and dedicated neurocritical care units (40%, n=36). Around 53% (n=47) reportedly used microsurgical repair to secure a ruptured intracerebral aneurysm, and endovascular repair was reported by 47% (n=42). The timing of aneurysm repair influenced the timing of EVD insertion in 63% (n=56) of responses. The freehand technique was the most common method for EVD insertion, utilized by 83% (n=74), followed by CT-guided in 25% (n=22) and ultrasound-guided techniques in 9% (n=8).

External ventricular drain insertion and maintenance practices

Non-antimicrobial-impregnated EVDs were the predominant choice, accounting for 90% (n=82) of cases, while antimicrobial-impregnated EVDs were reported by 10% (n=10). Most EVD insertions utilize commercially available systems, representing 85% (n=78), with indigenously designed systems employed in 15% (n=14), primarily due to cost constraints or local adaptations. For EVD insertion, a platelet count of greater than or equal to 100,000/cu.ml is required in 70% (n=64) of cases, while a threshold of greater than or equal to 75,000/cu.ml is used in 30% (n=28). The survey data highlights various practices regarding prophylactic antibiotic administration and venous thromboembolism (VTE) prophylaxis among patients with EVDs. A majority, 72% (n=64), administer prophylactic antibiotics, with the most common durations specified as one day (5%, n=5), three days (10%, n=9), five days (5%, n=5), seven days (15%, n=14), and 14 days (3%, n=3). An additional 6% (n=5) continue antibiotics for as long as the EVD is in place, and surgeon preference dictates antibiotic duration in 28% (n=25) of cases. For VTE chemoprophylaxis, about 30% (n=27) do not administer any chemical prophylaxis. Among those who do, low molecular weight heparin is the most frequently used agent, initiated at varying times post-EVD placement: within 12 hours (5%, n=5), 24 hours (40%, n=37), and more than 72 hours (3%, n=3). Unfractionated heparin is used less frequently, initiated within six hours (5%, n=5) and 12 hours (3%, n=3). EVD management responsibilities are divided between physicians (62%, n=55) and nurses (42%, n=37). Routine CSF checks are generally not conducted (92%, n=82). Those who perform routine CSF checks do so daily (1%, n=1) or weekly (3%, n=3), with clinical indications guiding these assessments.

Summary of external ventricular drain setting adjustments relative to aneurysm securement

The survey data indicate varied practices in adjusting EVD settings around the time of aneurysm securement. Consistent settings are maintained by 35% (n=14) of respondents, predominantly keeping the EVD at +15 cm H2O before and after securement, reflecting a strategy for stable ICP management throughout the perioperative period. Approximately 28% (n=12) of respondents report a reduction in EVD settings post-securement, typically lowering from +15 cm H2O to +10 cm H2O or from +20 cm H2O to +15 cm H2O, aiming to decrease intervention intensity once the aneurysm is secured. Increases in settings and complex, individualized adjustments are less common, comprising around 10% (n=4) of cases.

Clamp trial practices in neurosurgical external ventricular drain management

Clamp trials are reportedly routinely performed by 71% (n=63), indicating their standard role in managing patients with EVDs. The predominant method employed is slow clamping, which was reported by 45% (n=40). Rapid clamping is less common, reported by only 5% (n=5). Clamp trials were most frequently initiated after post-bleed day seven, reported by 84% (n=75). Additional reported criteria for starting clamp trials include the clarity of CSF and the reduction of hydrocephalus.

External ventricular drain management and intracranial pressure monitoring during patient transport

It was reported that 63% (n=56) of EVDs are predominantly kept closed during transport. Decisions to keep EVDs either open or closed were reportedly made on a case-by-case basis by 36% (n=32). ICP monitoring during transport was reported to be not routinely performed by 61% (n=54). In comparison, it is sometimes conducted by 26% (n=23) and always performed by 13% (n=11).

Quality improvement metrics for external ventricular drains

In our survey, a substantial majority of respondents, 73% (n=65), monitor for infections related to EVDs, making it the most commonly tracked QI metric. Conversely, hemorrhage and overdrainage are less frequently observed, with 29% (n=26) and 22% (n=20) tracking these metrics, respectively. Misplacement of EVDs is monitored by 30% (n=27).

Regional variability in external ventricular drain practices

Analysis of EVD practices across WHO regions revealed notable differences. Our analysis of the timing and setting of EVD insertion, management practices, and challenges in implementing EVD care protocols across various WHO regions (Table 3) yielded significant findings in a few areas. EVDs are typically inserted as soon as the diagnosis is made, with the timing of insertion showing statistically significant differences across regions (p=0.026). For instance, in East Asia & Pacific, 68% of EVDs are inserted immediately upon diagnosis, compared to 72% in South Asia and 45% in Latin America & Caribbean. Additionally, the management of EVD-related infections presents challenges, particularly regarding the prophylactic use of antibiotics after EVD insertion, which varies significantly across regions (p=0.003), with 74% adherence in East Asia & Pacific and 100% in Sub-Saharan Africa. Intrahospital transport of patients with EVDs is another area where practices significantly differ; decisions on whether to keep the EVD open or closed during transport showed variability (p=0.027), with 74% in Latin America & Caribbean keeping it closed, contrasting with 40% in South Asia. Moreover, ICP monitoring during intrahospital transport is notably different (p=0.008), where 88% of South Asian facilities do not monitor pressure compared to 47% in East Asia & Pacific.

**Table 3:** Comparison of reported practices by World Health Organization regions EVD: external ventricular drain; CSF: cerebrospinal fluid; WBC: while blood cell count; CRP: C-reactive protein; CT: computerized tomography of the brain; MRI: magnetic resonance imaging of the brain; aSAH: aneurysmal subarachnoid hemorrhage

	East Asia Pacific (N=19)	Europe & Central Asia (N=2)		Latin America & Caribbean (N=38)		South Asia (N=25)		Sub-Saharan Africa (N=5)		p-value
Indications for insertion of EVD										
Hydrocephalus	18 (95%)	2 (100%)		36 (95%)		25 (100%)		4 (80%)		0.3
Hunt and Hess grade	5 (26%)	0 (0%)		13 (34%)		8 (32%)		0 (0%)		0.7
World Federation of Neurological Surgeons grade	5 (26%)	0 (0%)		15 (39%)		4 (16%)		1 (20%)		0.4
Fisher or modified Fisher score	6 (32%)	1 (50%)		19 (50%)		10 (40%)		1 (20%)		0.7
Aneurysm location	2 (11%)	0 (0%)		4 (11%)		4 (16%)		1 (20%)		0.8
When are EVDs typically inserted?										0.026
As soon as the diagnosis is made	13 (68%)	0 (0%)		17 (45%)		18 (72%)		3 (60%)		
Delayed after the securement of the aneurysm	5 (26%)	2 (100%)		9 (24%)		5 (20%)		0 (0%)		
In the operating room, just before the start of the intervention	1 (5.3%)	0 (0%)		12 (32%)		2 (8.0%)		2 (40%)		
Who inserts EVDs at your hospital?										
Neurosurgery residents	9 (47%)	1 (50%)		20 (53%)		9 (36%)		3 (60%)		0.8
Neurosurgery fellows	6 (32%)	0 (0%)		7 (18%)		6 (24%)		1 (20%)		0.9
Attending neurosurgeon	14 (74%)	1 (50%)		29 (76%)		16 (64%)		4 (80%)		0.6
Where are EVDs routinely inserted?										
In the intensive care unit	3 (16%)	1 (50%)		8 (21%)		11 (44%)		2 (40%)		0.1
In the operating room	19 (100%)	2 (100%)		37 (97%)		22 (88%)		5 (100%)		0.3
In the emergency room	1 (5.3%)	0 (0%)		3 (7.9%)		3 (12%)		1 (20%)		0.6
On the floor/wards	0 (0%)	0 (0%)		0 (0%)		1 (4.0%)		0 (0%)		0.6
Where are patients with EVD managed at your hospital?										0.3
In a dedicated neurocritical care unit	10 (53%)	2 (100%)		13 (34%)		10 (40%)		1 (20%)		
In a general intensive care unit	9 (47%)	0 (0%)		23 (61%)		14 (56%)		3 (60%)		
On the floor/wards	0 (0%)	0 (0%)		2 (5.3%)		1 (4.0%)		1 (20%)		
Describe the prevalent technique to secure ruptured intracerebral aneurysms at your hospital										0.2
Clipping	13 (68%)	2 (100%)		19 (50%)		10 (40%)		3 (60%)		
Coiling	6 (32%)	0 (0%)		19 (50%)		15 (60%)		2 (40%)		
Describe the technique used to insert EVDs										
Freehand	13 (68%)	2 (100%)		34 (89%)		21 (84%)		4 (80%)		0.2
CT/MRI guided	8 (42%)	1 (50%)		8 (21%)		5 (20%)		0 (0%)		0.2
Ultrasound guided	1 (5.3%)	0 (0%)		3 (7.9%)		3 (12%)		1 (20%)		0.6
Describe the type of EVD inserted										0.4
Anti-microbial impregnated EVD	4 (21%)	0 (0%)		4 (11%)		5 (20%)		2 (40%)		
Non-antimicrobial impregnated EVD	15 (79%)	2 (100%)		30 (79%)		19 (76%)		2 (40%)		
Other	0 (0%)	0 (0%)		4 (11%)		1 (4.0%)		1 (20%)		
Describe the CSF system used										0.3
Commercially available system	15 (79%)	2 (100%)		34 (89%)		20 (80%)		3 (60%)		
Indigenously designed system	4 (21%)	0 (0%)		4 (11%)		5 (20%)		2 (40%)		
What platelet count is targeted prior to EVD insertion?										0.14
Greater than or equal to 100,000/cu.ml	13 (68%)	0 (0%)		24 (63%)		15 (60%)		3 (60%)		
Greater than or equal to 75,000/cu.ml	4 (21%)	1 (50%)		11 (29%)		10 (40%)		1 (20%)		
None	2 (11%)	1 (50%)		1 (2.6%)		0 (0%)		1 (20%)		
Other	0 (0%)	0 (0%)		2 (5.3%)		0 (0%)		0 (0%)		
How do you typically prevent and monitor for EVD-related infections?										
Use of anti-microbial impregnated catheters	4 (21%)	0 (0%)		5 (13%)		5 (20%)		1 (20%)		>0.9
Sterile technique while inserting	15 (79%)	2 (100%)		35 (92%)		25 (100%)		4 (80%)		0.092
One dose of antibiotic before insertion of the EVD	12 (63%)	2 (100%)		23 (61%)		13 (52%)		4 (80%)		0.7
Tunneling of the catheter	9 (47%)	2 (100%)		13 (34%)		11 (44%)		1 (20%)		0.3
CSF sampling is strictly limited to when clinically indicated	10 (53%)	2 (100%)		26 (68%)		19 (76%)		3 (60%)		0.5
Continuous monitoring for signs of infection (fever, WBC count, CRP)	17 (89%)	2 (100%)		28 (74%)		21 (84%)		5 (100%)		0.7
At your hospital, do you use prophylactic antibiotics after EVD insertion?	14 (74%)	1 (50%)		14 (37%)		15 (60%)		5 (100%)		0.003
When is venous thromboprophylaxis initiated after EVD insertion?										
12 hours	3 (16%)	1 (50%)		5 (13%)		6 (24%)		0 (0%)		
24 hours	1 (5.3%)	0 (0%)		15 (39%)		14 (56%)		0 (0%)		
4 hours	0 (0%)	0 (0%)		1 (2.6%)		0 (0%)		0 (0%)		
6 hours	1 (5.3%)	1 (50%)		5 (13%)		0 (0%)		1 (20%)		
Other	3 (16%)	0 (0%)		4 (11%)		2 (8.0%)		4 (80%)		
We do not administer chemical thromboprophylaxis	11 (58%)	0 (0%)		8 (21%)		3 (12%)		0 (0%)		
Drug of choice for venous thromboprophylaxis										0.003
Low molecular weight heparin	7 (37%)	2 (100%)		31 (82%)		18 (72%)		3 (60%)		
Unfractionated heparin	2 (11%)	0 (0%)		2 (5.3%)		4 (16%)		2 (40%)		
We do not use any chemical prophylaxis	10 (53%)	0 (0%)		5 (13%)		3 (12%)		0 (0%)		
Who manages EVDs at your hospital?										0.2
Nurses	13 (68%)	0 (0%)		22 (58%)		19 (76%)		2 (40%)		
Physicians	6 (32%)	2 (100%)		16 (42%)		6 (24%)		3 (60%)		
EVD-associated infection surveillance										
Daily CSF is drawn for infection surveillance	1 (5.3%)	0 (0%)		6 (16%)		1 (4.0%)		0 (0%)		0.6
CSF is drawn weekly for infection surveillance	5 (26%)	1 (50%)		6 (16%)		3 (12%)		1 (20%)		0.4
CSF is collected more than once a week for infection surveillance	2 (11%)	1 (50%)		7 (18%)		2 (8.0%)		1 (20%)		0.4
CSF is not routinely collected for infection surveillance	11 (58%)	0 (0%)		19 (50%)		19 (76%)		3 (60%)		0.2
Setting of EVD prior to the securement of the aneurysm										
+ 10 cm H20	1 (5.3%)	0 (0%)		3 (7.9%)		3 (12%)		0 (0%)		
+ 15 cm H20	11 (58%)	0 (0%)		21 (55%)		11 (44%)		3 (60%)		
+ 20 cm H20	4 (21%)	2 (100%)		9 (24%)		3 (12%)		2 (40%)		
+ 5 cm H20	1 (5.3%)	0 (0%)		2 (5.3%)		5 (20%)		0 (0%)		
Other	2 (11%)	0 (0%)		3 (7.9%)		3 (12%)		0 (0%)		
Setting of EVD after securement of the aneurysm										
+ 10 cm H20	8 (42%)	1 (50%)		5 (13%)		10 (40%)		3 (60%)		
+ 15 cm H20	8 (42%)	1 (50%)		24 (63%)		7 (28%)		1 (20%)		
+ 20 cm H20	0 (0%)	0 (0%)		2 (5.3%)		1 (4.0%)		1 (20%)		
+ 5 cm H20	1 (5.3%)	0 (0%)		5 (13%)		3 (12%)		0 (0%)		
Other	2 (11%)	0 (0%)		2 (5.3%)		4 (16%)		0 (0%)		
How are EVDs managed?										0.7
CSF is drained based on ICP monitoring	5 (26%)	1 (50%)		18 (47%)		6 (24%)		2 (40%)		
CSF is continuously drained	10 (53%)	1 (50%)		16 (42%)		14 (56%)		3 (60%)		
CSF is intermittently drained	4 (21%)	0 (0%)		3 (7.9%)		5 (20%)		0 (0%)		
Other	0 (0%)	0 (0%)		1 (2.6%)		0 (0%)		0 (0%)		
All patients with EVDs undergo a clamp trial prior to permanent CSF diversion device insertion	16 (84%)	2 (100%)		29 (78%)		20 (80%)		4 (80%)		>0.9
EVD clamp trial is slow (slow clamp trial (+10, +20, clamp with 24-hour intervals)	14 (74%)	0 (0%)		21 (55%)		14 (56%)		4 (80%)		0.2
EVDs clamp trial is rapid (rapid clamp trial (+10, clamp)	4 (21%)	2 (100%)		13 (34%)		8 (32%)		0 (0%)		0.13
When is the EVD clamp trial generally held?										0.2
After 7 days	17 (89%)	2 (100%)		39 (95%)		20 (80%)		4 (80%)		
After 5 days	0 (0%)	0 (0%)		0 (0%)		1 (4.0%)		0 (0%)		
After CSF becomes non-hemorrhagic	0 (0%)	0 (0%)		1 (2.6%)		0 (0%)		0 (0%)		
As early as symptoms resolve and output starts to decrease	0 (0%)	0 (0%)		0 (0%)		0 (0%)		1 (20%)		
Clinical improvement, no complication of SAH, no CSF drainage	0 (0%)	0 (0%)		0 (0%)		1 (4.0%)		0 (0%)		
How are EVDs managed during intrahospital transport?										0.027
Always kept close to CSF drainage	13 (68%)	1 (50%)		28 (74%)		10 (40%)		4 (80%)		
Always kept open to CSF drainage	1 (5.3%)	0 (0%)		0 (0%)		0 (0%)		0 (0%)		
Open or closed to CSF drainage is on a case-by-case basis	5 (26%)	1 (50%)		10 (26%)		15 (60%)		1 (20%)		
Is intracranial pressure monitored during intrahospital transport?									0.008	
Never	9 (47%)	1 (50%)		18 (47%)		22 (88%)		4 (80%)		
Yes: always	1 (5.3%)	0 (0%)		8 (21%)		1 (4.0%)		1 (20%)		
Yes: sometimes	9 (47%)	1 (50%)		11 (29%)		2 (8.0%)		0 (0%)		
Describe challenges that you observe at your hospital										
Availability of EVD catheters	9 (47%)	0 (0%)		14 (37%)		3 (12%)		4 (80%)		0.008
Availability of neuroimaging	5 (26%)	0 (0%)		8 (21%)		3 (12%)		3 (60%)		0.2
Training of staff	5 (26%)	0 (0%)		10 (26%)		8 (32%)		3 (60%)		0.5
EVD-associated infection	3 (16%)	2 (100%)		17 (45%)		13 (52%)		3 (60%)		0.029
EVD-associated hemorrhage	1 (5.3%)	0 (0%)		9 (24%)		0 (0%)		0 (0%)		0.056
Misplaced catheter	1 (5.3%)	0 (0%)		11 (29%)		4 (16%)		1 (20%)		0.3
Describe quality metrics tracked at your hospital										
EVD-associated infection	14 (74%)	2 (100%)		27 (71%)		18 (72%)		2 (40%)		0.6
EVD-associated hemorrhage	4 (21%)	1 (50%)		14 (37%)		5 (20%)		1 (20%)		0.5
CSF over drainage	3 (16%)	2 (100%)		9 (24%)		4 (16%)		2 (40%)		0.1
Misplaced catheter	4 (21%)	1 (50%)		14 (37%)		5 (20%)		1 (20%)		0.4

Analysis of external ventricular drain practices across country types

Our study identified statistically significant variations in the practices of EVD management across income groups (Table 4). Who inserts EVDs at your hospital shows substantial differences (p=0.022), with neurosurgery residents performing the procedure in 75% of cases in low-income settings, compared to 29% in lower-middle-income and 56% in upper-middle-income countries. The location of EVD insertion in the intensive care unit also varied significantly (p=0.027), occurring in 50% of low-income, 42% of lower-middle-income, and only 19% of upper-middle-income countries. Regarding the type of EVD inserted, a significant difference was observed (p=0.05), with antimicrobial-impregnated EVDs used in 50% of low-income settings, compared to 23% in lower-middle-income and 11% in upper-middle-income countries. Prophylactic antibiotic use post-EVD insertion differed significantly (p=0.019), with 100% usage in low-income countries, 68% in lower-middle-income countries, and 44% in upper-middle-income countries. Another significant finding was the setting of EVD after the securement of the aneurysm (p=0.032), indicating varying practices in fluid management post-procedure. Lastly, the EVD clamp trial timing also showed significant variation (p=0.042), particularly with 96% of upper-middle-income settings performing the clamp after seven days compared to 77% in lower-middle-income settings and 75% in low-income settings.

**Table 4:** Comparison of reported practices by income groups EVD: external ventricular drain; CSF: cerebrospinal fluid; WBC: while blood cell count; CRP: C-reactive protein; CT: computerized tomography of the brain; MRI: magnetic resonance imaging of the brain; aSAH: aneurysmal subarachnoid hemorrhage

	Low income N=4	Lower-middle=income N=31	Upper-middle-income N=54	p-value
Indications for insertion of EVD				
Hydrocephalus	3 (75%)	30 (97%)	52 (96%)	0.2
Hunt and Hess grade	0 (0%)	10 (32%)	16 (30%)	0.5
World Federation of Neurological Surgeons grade	1 (25%)	5 (16%)	19 (35%)	0.2
Fisher or modified Fisher score	1 (25%)	11 (35%)	25 (46%)	0.5
Aneurysm location	1 (25%)	6 (19%)	4 (7.4%)	0.2
When are EVDs typically inserted?				0.074
As soon as the diagnosis is made	2 (50%)	22 (71%)	27 (50%)	
Delayed after the securement of the aneurysm	0 (0%)	7 (23%)	14 (26%)	
In the operating room, just before the start of the intervention	2 (50%)	2 (6.5%)	13 (24%)	
Who inserts EVDs at your hospital?				
Neurosurgery residents	3 (75%)	9 (29%)	30 (56%)	0.022
Neurosurgery fellows	1 (25%)	9 (29%)	10 (19%)	0.5
Attending neurosurgeon	3 (75%)	21 (68%)	40 (74%)	0.8
Where are EVDs routinely inserted?				
In the intensive care unit	2 (50%)	13 (42%)	10 (19%)	0.027
In the operating room	4 (100%)	28 (90%)	53 (98%)	0.3
In the emergency room	1 (25%)	3 (9.7%)	4 (7.4%)	0.4
On the floor/wards	0 (0%)	1 (3.2%)	0 (0%)	0.4
Where are patients with EVD managed at your hospital?				0.4
In a dedicated neurocritical care unit	1 (25%)	14 (45%)	21 (39%)	
In a general intensive care unit	2 (50%)	16 (52%)	31 (57%)	
On the floor/wards	1 (25%)	1 (3.2%)	2 (3.7%)	
Describe the prevalent technique to secure ruptured intracerebral aneurysms at your hospital				>0.9
Clipping	2 (50%)	17 (55%)	28 (52%)	
Coiling	2 (50%)	14 (45%)	26 (48%)	
Describe the technique used to insert EVDs				
Freehand	4 (100%)	23 (74%)	47 (87%)	0.3
CT/MRI guided	0 (0%)	9 (29%)	13 (24%)	0.6
Ultrasound guided	0 (0%)	5 (16%)	3 (5.6%)	0.2
Describe the type of EVD inserted				0.05
Anti-microbial impregnated EVD	2 (50%)	7 (23%)	6 (11%)	
Non-antimicrobial impregnated EVD	1 (25%)	23 (74%)	44 (81%)	
Other	1 (25%)	1 (3.2%)	4 (7.4%)	
Describe the CSF system used				0.2
Commercially available system	2 (50%)	26 (84%)	46 (85%)	
Indigenously designed system	2 (50%)	5 (16%)	8 (15%)	
What platelet count is targeted prior to EVD insertion?				0.4
Greater than or equal to 100,000/cu.ml	2 (50%)	18 (58%)	35 (65%)	
Greater than or equal to 75,000/cu.ml	1 (25%)	12 (39%)	14 (26%)	
None	1 (25%)	1 (3.2%)	3 (5.6%)	
Other	0 (0%)	0 (0%)	2 (3.7%)	
How do you typically prevent and monitor for EVD-related infections?				
Use of anti-microbial impregnated catheters	1 (25%)	6 (19%)	8 (15%)	>0.9
Sterile technique while inserting	4 (100%)	29 (94%)	48 (89%)	0.8
One dose of antibiotic before insertion of the EVD	4 (100%)	16 (52%)	34 (63%)	0.2
Tunneling of the catheter	1 (25%)	12 (39%)	23 (43%)	0.8
CSF sampling is strictly limited to when clinically indicated	3 (75%)	22 (71%)	35 (65%)	0.9
Continuous monitoring for signs of infection (fever, WBC count, CRP)	4 (100%)	26 (84%)	43 (80%)	>0.9
At your hospital, do you use prophylactic antibiotics after EVD insertion?	4 (100%)	21 (68%)	24 (44%)	0.019
When is venous thromboprophylaxis initiated after EVD insertion?				0.018
12 hours	0 (0%)	8 (26%)	7 (13%)	
24 hours	0 (0%)	13 (42%)	17 (31%)	
4 hours	0 (0%)	0 (0%)	1 (1.9%)	
6 hours	1 (25%)	0 (0%)	7 (13%)	
Other	3 (75%)	3 (9.7%)	7 (13%)	
We do not administer chemical thromboprophylaxis	0 (0%)	7 (23%)	15 (28%)	
Drug of choice for venous thromboprophylaxis				0.088
Low molecular weight heparin	2 (50%)	19 (61%)	40 (74%)	
Unfractionated heparin	2 (50%)	5 (16%)	3 (5.6%)	
We do not use any chemical prophylaxis	0 (0%)	7 (23%)	11 (20%)	
Who manages EVDs at your hospital?				0.5
Nurses	2 (50%)	22 (71%)	32 (59%)	
Physicians	2 (50%)	9 (29%)	22 (41%)	
EVD-associated infection surveillance				
Daily CSF is drawn for infection surveillance	0 (0%)	2 (6.5%)	6 (11%)	0.6
CSF is drawn weekly for infection surveillance	1 (25%)	6 (19%)	9 (17%)	0.7
CSF is collected more than once a week for infection surveillance	0 (0%)	4 (13%)	9 (17%)	0.9
CSF is not routinely collected for infection surveillance	3 (75%)	19 (61%)	30 (56%)	0.7
Setting of EVD prior to the securement of the aneurysm				0.3
+ 10 cm H20	0 (0%)	4 (13%)	3 (5.6%)	
+ 15 cm H20	3 (75%)	15 (48%)	28 (52%)	
+ 20 cm H20	1 (25%)	3 (9.7%)	16 (30%)	
+ 5 cm H20	0 (0%)	5 (16%)	3 (5.6%)	
Other	0 (0%)	4 (13%)	4 (7.4%)	
Setting of EVD after securement of the aneurysm				0.032
+ 10 cm H20	2 (50%)	13 (42%)	12 (22%)	
+ 15 cm H20	1 (25%)	8 (26%)	32 (59%)	
+ 20 cm H20	1 (25%)	1 (3.2%)	2 (3.7%)	
+ 5 cm H20	0 (0%)	4 (13%)	5 (9.3%)	
Other	0 (0%)	5 (16%)	3 (5.6%)	
How are EVDs managed?				0.7
CSF is drained based on ICP Monitoring	1 (25%)	9 (29%)	22 (41%)	
CSF is continuously drained	3 (75%)	16 (52%)	25 (46%)	
CSF is intermittently drained	0 (0%)	6 (19%)	6 (11%)	
Other	0 (0%)	0 (0%)	1 (1.9%)	
All patients with EVDs undergo a clamp trial prior to permanent CSF diversion device insertion	3 (75%)	24 (77%)	44 (83%)	0.6
EVD clamp trial is slow (slow clamp trial (+10, +20, clamp with 24-hour intervals)	3 (75%)	16 (52%)	34 (63%)	0.6
EVDs clamp trial is rapid (rapid clamp trial (+10, clamp)	0 (0%)	10 (32%)	17 (31%)	0.6
When is the EVD clamp trial generally held?				0.042
After 7 days	3 (75%)	24 (77%)	52 (96%)	
After 5 days	0 (0%)	1 (3.2%)	0 (0%)	
After CSF becomes non-hemorrhagic	0 (0%)	1 (3.2%)	0 (0%)	
As early as symptoms resolve, and output starts to decrease	1 (25%)	0 (0%)	0 (0%)	
Clinical improvement, no complication of SAH, no CSF drainage	0 (0%)	1 (3.2%)	0 (0%)	
How are EVDs managed during intrahospital transport?				0.14
Always kept close to CSF drainage	3 (75%)	15 (48%)	38 (70%)	
Always kept open to CSF drainage	0 (0%)	0 (0%)	1 (1.9%)	
Open or closed to CSF drainage is on a case-by-case basis	1 (25%)	16 (52%)	15 (28%)	
Is intracranial pressure monitored during intrahospital transport?				0.091
Never	3 (75%)	24 (77%)	27 (50%)	
Yes: always	1 (25%)	1 (3.2%)	9 (17%)	
Yes: sometimes	0 (0%)	6 (19%)	17 (31%)	
Describe challenges that you observe at your hospital				
Availability of EVD catheters	4 (100%)	5 (16%)	21 (39%)	0.001
Availability of neuroimaging	3 (75%)	5 (16%)	11 (20%)	0.047
Training of staff	3 (75%)	9 (29%)	14 (26%)	0.14
EVD-associated infection	2 (50%)	16 (52%)	20 (37%)	0.4
EVD-associated hemorrhage	0 (0%)	1 (3.2%)	9 (17%)	0.2
Misplaced catheter	0 (0%)	6 (19%)	11 (20%)	>0.9
Describe quality metrics tracked at your hospital				
EVD-associated infection	1 (25%)	22 (71%)	40 (74%)	0.14
EVD-associated hemorrhage	1 (25%)	6 (19%)	18 (33%)	0.4
CSF over drainage	2 (50%)	5 (16%)	13 (24%)	0.3
Misplaced catheter	0 (0%)	6 (19%)	19 (35%)	0.2

Challenges in implementing subarachnoid hemorrhage and external ventricular drain guidelines and quality improvement opportunities related to external ventricular drain care

**Figure 2:**
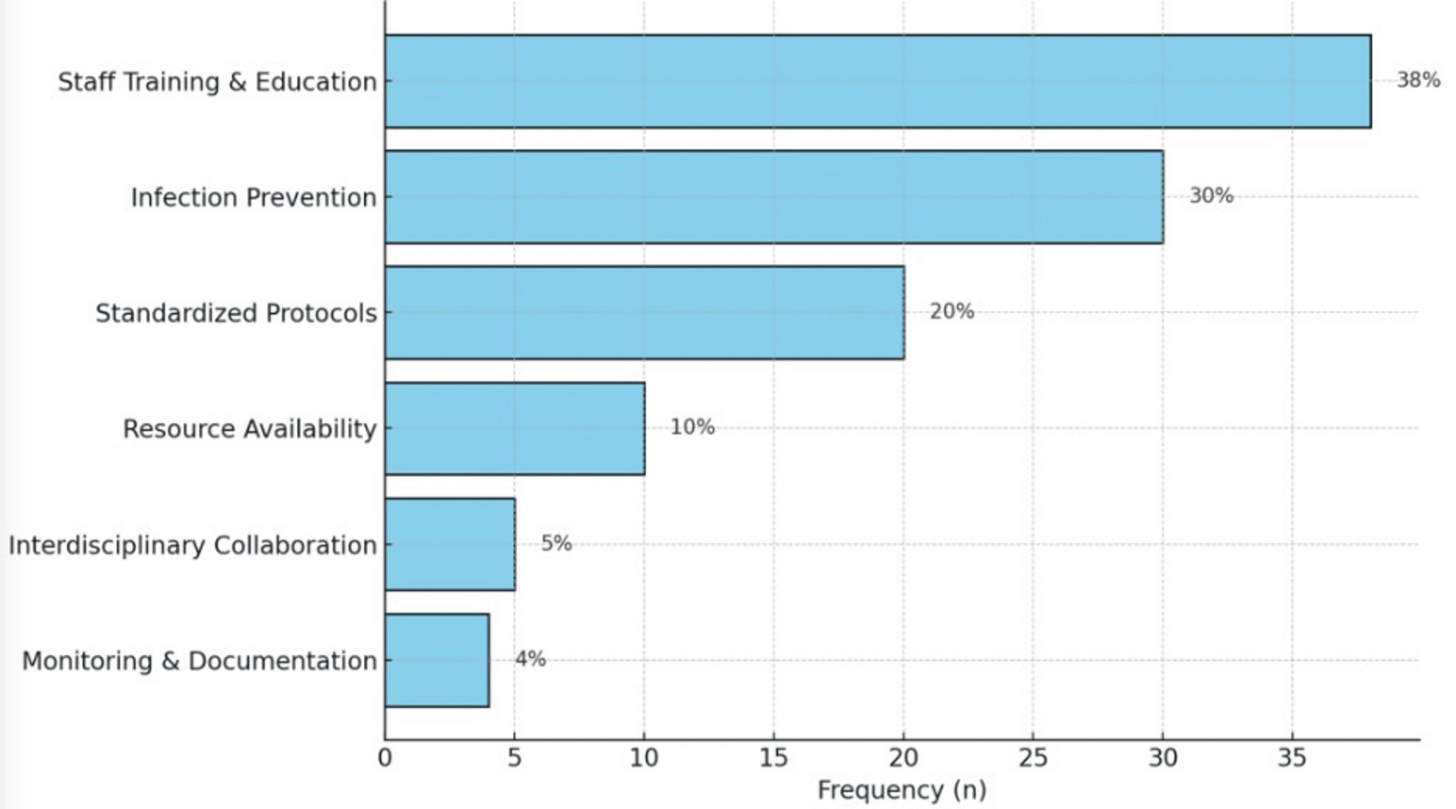
Challenges in implementing aSAH and EVD guidelines and quality improvement opportunities aSAH: aneurysmal subarachnoid hemorrhage; EVD: external ventricular drain

We used free text responses to create themes. A thematic analysis of challenges reported by participants in implementing guidelines for EVD management revealed a range of obstacles. Predominantly, 35% (n=28) of respondents identified a significant lack of formal training and education for staff involved in EVD handling, including insufficient knowledge in areas such as CSF examination and clamping of EVDs. About 30% (n=24) of the responses highlighted resource limitations (shortages of EVDs, monitors, and neurosurgeons). Infection control challenges were noted by 20% (n=16) of participants, who reported challenges in maintaining sterile techniques and managing high rates of ventriculitis. Interdepartmental coordination issues were mentioned by 10% (n=8). Finally, about 5% (n=4) indicated problems with the variability of guidelines and staying updated with their changes. These themes are summarized in Figure 2. 

Alignment of prevalent practices with published guidelines

**Figure 3:**
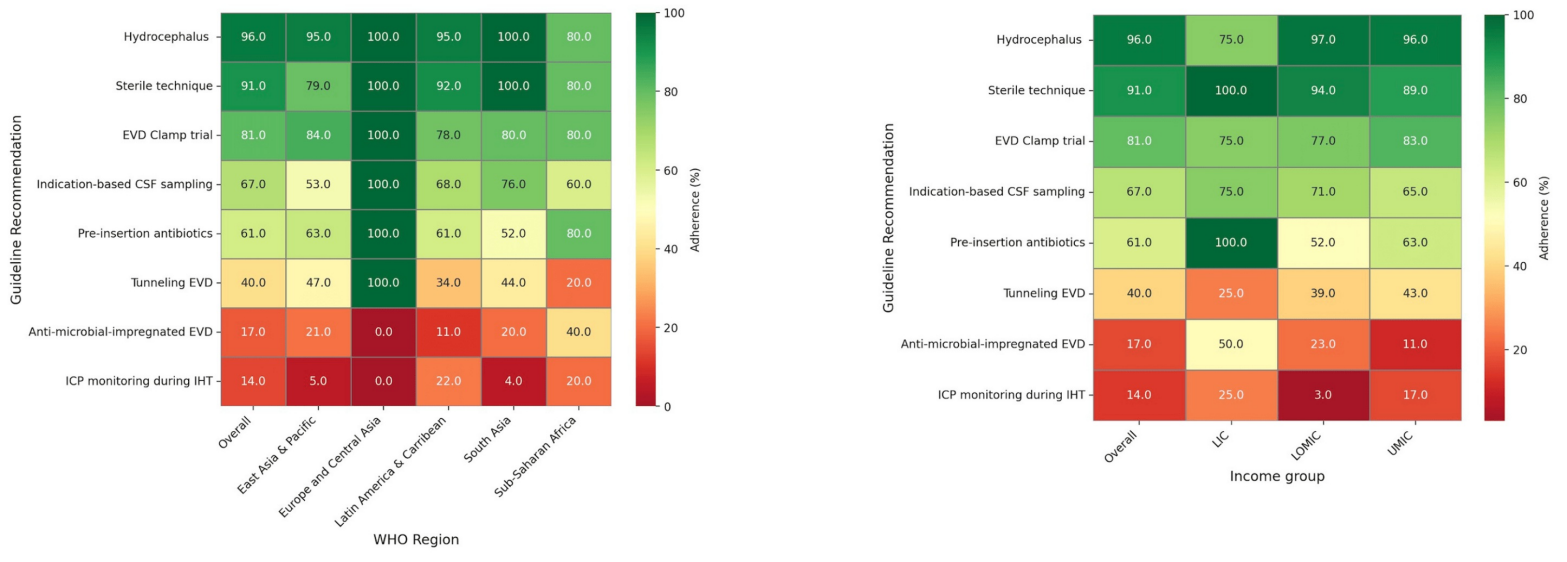
Adherence to guideline recommendations (variability in EVD practices for aSAH management by WHO regions and income groups) EVD: external ventricular drain; aSAH: aneurysmal subarachnoid hemorrhage; WHO: World Health Organization

The heatmap (**Figure 3**) illustrates adherence rates to guideline recommendations (overall and by WHO regions) and income groups.

Adherence by the WHO regions

For hydrocephalus management, 100% adherence was observed in Europe, Central Asia, and South Asia, while East Asia & Pacific and Latin America & Caribbean reported slightly lower rates at 95%. Sub-Saharan Africa had the lowest adherence at 80%. EVD clamp trial adherence was perfect in Europe & Central Asia at 100% and generally consistent across other regions, ranging from 78% to 80%. Intrahospital transfer ICP monitoring demonstrated significant variability, with no adherence in Europe & Central Asia, low adherence in East Asia & Pacific and South Asia at 5% and 4%, respectively, and relatively higher adherence in Sub-Saharan Africa at 20% and Latin America & Caribbean at 21%. Indication-based CSF sampling showed perfect adherence in Europe & Central Asia at 100%. At the same time, other regions varied, with Latin America & Caribbean at 68%, South Asia at 76%, East Asia & Pacific at 53%, and Sub-Saharan Africa at 60%. The use of anti-microbial-impregnated EVDs showed no adherence in Europe & Central Asia, 21% in East Asia & Pacific, and varying levels in other regions, with Latin America & Caribbean at 11%, South Asia at 20%, and Sub-Saharan Africa at 40%. EVD tunneling practices were 100% adherent in Europe & Central Asia, with other regions showing lower adherence: East Asia and Pacific at 47%, Latin America & Caribbean at 34%, South Asia at 44%, and Sub-Saharan Africa at 20%. Pre-insertion antibiotics also demonstrated perfect adherence in Europe & Central Asia at 100%, with lower rates in East Asia & the Pacific at 63%, Latin America & the Caribbean at 61%, South Asia at 52%, and Sub-Saharan Africa at 80%. Sterile techniques achieved 100% adherence in Europe, Central Asia, and South Asia, 92% in Latin America & Caribbean, 79% in East Asia & Pacific, and 80% in Sub-Saharan Africa.

Adherence by country type

In managing hydrocephalus, adherence was high in lower-middle-income countries (LMICs) and upper-middle-income countries (UMICs) at 97% and 96%, respectively, compared to 75% in low-income countries (LICs). For EVD clamp trials, adherence was reasonably consistent across all income groups, recorded at 75% in LICs, 77% in LMICs, and 83% in UMICs. Intrahospital transfer ICP monitoring adherence was significantly lower, especially in LMICs at only 3%, with LICs at 25%, and UMICs at 17%. Indication-based CSF sampling showed the highest adherence in LICs at 75%, with LMICs and UMICs showing slightly lower rates at 71% and 65%, respectively. The use of anti-microbial-impregnated EVDs varied greatly, with LICs at 50% and much lower in LMICs and UMICs at 23% and 11%. The EVD tunneling practices showed an adherence of 25% in LICs, with higher rates in LMICs and UMICs at 39% and 43%. The administration of pre-insertion antibiotics demonstrated perfect adherence in LICs at 100%, but significantly lower in LMICs and UMICs at 52% and 63%. Sterile techniques were very high across all regions, peaking in LICs at 100%, with 94% in LMICs and 89% in UMICs.

Alternate methods to external ventricular drains

Alternative methods for EVDs in resource-limited settings include the use of nasogastric (NG) tubes, size 6 (n=2), or 8-10 French feeding tubes (n=2). Additionally, urinary catheters (n=2) are considered alternative drainage systems.

Equipment cost

The reported costs of EVDs and CSF collection systems varied widely across surveyed LMIC hospitals. In Brazil, an EVD was reported to cost approximately R$ 720,00, while in Colombia, prices ranged from 300,114 COP (~USD 75) to 1,156,670 COP (~USD 290), with some models subject to additional taxation. In Vietnam, EVD sets were priced between 170 and 200 USD (4,000,000 VND), with CSF collection bags costing an extra 20 USD (500,000 VND). Some hospitals relied on lower-cost alternatives from regional manufacturers when standard supplies were unavailable. In Ecuador, EVD catheters were reported at USD 380, while alternative systems reached up to USD 1,500. In Nepal, EVD systems cost NRs 4,000-5,000 (~USD 30-40), with procedural insertion costs ranging from NRs 12,000-15,000 (~USD 90-115). Additionally, in India, the cost of a CSF collection system was reported at 5,000 INR (~USD 60).

## Discussion

In LMICs, this study highlights the current practices, challenges, and opportunities for QI in managing EVDs in patients with aSAH. The main findings are variability in EVD insertion and maintenance practices, particularly in reducing infection risks, mitigating intrahospital transport-associated complications, and adhering to guidelines, and inconsistent tracking of QI metrics, such as hemorrhage and overdrainage. These findings underscore the need for tailored interventions to enhance patient safety and care delivery, providing a foundation for actionable QI initiatives.

The study emphasizes the prominence of hydrocephalus as the primary determinant for EVD insertion, with practices primarily aligning with AHA guidelines. However, regional variability in timing reflects a lack of standardization. For instance, South Asia (72%) and East Asia and the Pacific (68%) preferred immediate insertion. At the same time, Latin America & the Caribbean showed a more cautious approach, with one in two hospitals reporting delaying insertion until after aneurysm securement. These differences may stem from resource constraints, cultural variations in clinical decision-making, or differences in training and institutional protocols.

Using indigenously designed EVD systems, such as feeding tubes, in resource-limited regions like South Asia and Sub-Saharan Africa highlights cost-saving strategies. Still, it raises questions about the consistency of manufacturing standards. In contrast, commercially available systems were predominantly used in Latin America and the Caribbean, reflecting better access to standardized equipment. These disparities underline the need for localized approaches that balance affordability, safety, and efficacy.

Antimicrobial-impregnated catheters and pre-procedure antibiotics remain inconsistent. The low usage of antimicrobial catheters (17%), despite their proven efficacy in reducing ventriculitis [10,11,12], reflects cost barriers and limited access. Future research should focus on evaluating antimicrobial catheters' economic and clinical impacts in resource-constrained settings to guide adoption. The costs of EVDs and CSF collection systems highlight substantial financial barriers in LMICs. The reliance on lower-cost alternatives from regional manufacturers in certain hospitals underscores the resource-driven nature of procurement decisions, potentially affecting the consistency of care. These findings reveal stark disparities in neurocritical care accessibility, where the availability of life-saving interventions is not solely dictated by clinical need but by economic constraints, import taxation, and institutional purchasing power. Addressing these disparities requires strategic policies aimed at cost-effective procurement, subsidy programs, and standardization efforts to ensure equitable access to neurosurgical care across diverse healthcare settings. Additionally, variability in sterile techniques and pre-procedure antibiotics highlights gaps in training and access to aseptic supplies, further exacerbating infection risks.

Intrahospital transport practices reveal additional safety concerns, with most respondents reporting closed EVD systems during transport and inconsistent ICP monitoring. While these practices may minimize the risk of overdrainage, they raise concerns about ICP elevation, especially during the early phases of aSAH [13,14,15]. Regions such as South Asia and sub-Saharan Africa reported the lowest rates of ICP monitoring during transport, reflecting resource limitations. Adopting cost-effective monitoring solutions, as emphasized in the SNACC framework, could address these gaps.

Routine tracking of infection-related metrics demonstrates an encouraging awareness of QI, yet other complications, like hemorrhage and overdrainage, are less frequently monitored. Comprehensive QI programs that expand metric tracking to include these parameters could provide a holistic view of EVD safety and effectiveness.

Regional differences in guideline adherence further highlight the challenges of implementing uniform recommendations in diverse healthcare settings. For instance, South Asia demonstrated higher adherence to sterile techniques, while Sub-Saharan Africa reported higher usage of antimicrobial catheters, possibly due to targeted funding. These findings suggest that adapting global guidelines to regional contexts and emphasizing training and capacity building are crucial for improving outcomes. Initiatives like the SNACC EVD Safety Campaign [16], which focuses on scalable interventions, routine audits, and multidisciplinary training, offer a practical framework for addressing these gaps. The EVD Safety Campaign could serve as a model to promote awareness and standardized practices across LMICs. The SNACC EVD Safety Campaign, focusing on education, infection prevention, and interdisciplinary collaboration, offers a practical framework for addressing some of these gaps in LMICs. By promoting a culture of QI and encouraging the adoption of standardized care bundles, the campaign can catalyze improvements in EVD safety and outcomes in LMICs.

Education and training emerged as critical areas for improvement. The survey highlighted a perceived or self-reported lack of comprehensive training for neurosurgeons, intensivists, and nurses in EVD management. Targeted programs, including workshops and simulation-based training, can build local capacity and foster adherence to best practices. Emphasizing the role of nurses in EVD management, particularly in ICP monitoring and transport practices, could enhance safety and care delivery. Expanding access to standardized protocols, infection prevention strategies, and QI initiatives can align practices with global guidelines while addressing local constraints.

Strengths and limitations

Surveys are valuable for quickly catching prevailing practices, especially in resource-limited settings. They allow for broad data collection on diverse clinical practices and decision-making. However, surveys are inherently limited by potential response bias and self-reported inaccuracies, which may not fully capture the complexity of real-world practices. Europe & Central Asia and Sub-Saharan Africa were underrepresented in the survey. Furthermore, this study's reliance on LMIC respondents limits generalizability to high-income countries but ensures a focused evaluation of resource-constrained settings. Despite these limitations, the findings underscore critical areas for intervention and provide a roadmap for integrating evidence-based practices into LMIC healthcare systems.

## Conclusions

Significant gaps in the care associated with EVD in LMICs underscore a pressing need for QI initiatives that are not only tailored but are also sensitive to the unique challenges of these regions. These gaps are prevalent across diverse healthcare settings within LMICs, reflecting stark discrepancies in practices that often stem from differences in resource availability, the level of training among healthcare professionals, and the degree of adherence to global clinical guidelines. Campaigns like the SNACC EVD Safety Campaign exemplify strategic efforts to bridge these gaps through education and standardization of practices. By providing structured training modules, up-to-date procedural guidelines, and resources for effective EVD management, such initiatives can significantly elevate the level of care. Moreover, these campaigns promote a culture of safety and continuous improvement through regular audits and feedback mechanisms, which are essential for sustaining change. However, for these initiatives to be truly effective in LMICs, they must be adaptable to the constraints of these settings. This means that QI practices must be scalable and flexible, allowing for modifications based on available resources, cultural contexts, and existing healthcare infrastructure. For example, while using antimicrobial-impregnated catheters is recommended to reduce infection rates, their high cost may not be justifiable or feasible in all LMIC settings. Instead, alternative strategies such as rigorous training in aseptic insertion techniques might provide a more cost-effective solution. By addressing these elements, QI initiatives can substantially improve EVD care in LMICs, ultimately leading to better patient outcomes and more efficient healthcare systems. Such efforts underline the importance of global health equity and the need for continued investment in health systems strengthening in resource-limited settings.
